# Hybridization between two cestode species and its consequences for intermediate host range

**DOI:** 10.1186/1756-3305-6-33

**Published:** 2013-02-07

**Authors:** Tina Henrich, Daniel P Benesh, Martin Kalbe

**Affiliations:** 1 Department of Evolutionary Ecology, Max Planck Institute for Evolutionary, Biology, August-Thienemann-Strasse 2, Plön, 24306, Germany

## Abstract

**Background:**

Many parasites show an extraordinary degree of host specificity, even though a narrow range of host species reduces the likelihood of successful transmission. In this study, we evaluate the genetic basis of host specificity and transmission success of experimental F_1_ hybrids from two closely related tapeworm species (*Schistocephalus solidus* and *S. pungitii*), both highly specific to their respective vertebrate second intermediate hosts (three- and nine-spined sticklebacks, respectively).

**Methods:**

We used an *in vitro* breeding system to hybridize *Schistocephalus solidus* and *S. pungitii*; hybridization rate was quantified using microsatellite markers. We measured several fitness relevant traits in pure lines of the parental parasite species as well as in their hybrids: hatching rates, infection rates in the copepod first host, and infection rates and growth in the two species of stickleback second hosts.

**Results:**

We show that the parasites can hybridize in the *in vitro* system, although the proportion of self-fertilized offspring was higher in the heterospecific breeding pairs than in the control pure parental species. Hybrids have a lower hatching rate, but do not show any disadvantages in infection of copepods. In fish, hybrids were able to infect both stickleback species with equal frequency, whereas the pure lines were only able to infect their normal host species.

**Conclusions:**

Although not yet documented in nature, our study shows that hybridization in *Schistocephalus* spp. is in principle possible and that, in respect to their expanded host range, the hybrids are fitter. Further studies are needed to find the reason for the maintenance of the species boundaries in wild populations.

## Background

In interaction with their host organisms, many parasite taxa show an extraordinary degree of specificity, which is often regarded as indication of a long co-evolutionary history. In fact, parasites with a rather narrow range of suitable host species have been shown to be better adapted to sympatric host populations than generalist parasites [1,2]. However, the actual advantage of being restricted to only one or very few host species is still elusive. Particularly for parasites with complex life cycles, a narrow host range can be very disadvantageous since it decreases the probability for transmission when suitable host species are rare. Therefore, a good strategy for a parasite would be to become optimally adapted to one host species, but capable of a host-switch to avoid extinction when under changing ecological conditions the specific host disappears.

One possibility for a rather fast expansion of the host range could be the introgression of host compatibility genes by hybridization between closely related parasites species [3]. Furthermore, this might also be a way to escape extinction, since specialization has been suggested as a one-way street [4,5]. Such a scenario is particularly conceivable in macroparasites with complex life cycles, where two parental species are highly specific to different intermediate hosts, but share a common final host where sexual reproduction takes place.

In all major taxa of helminth parasites, hybridization has been found in nature or been demonstrated between sympatric species in laboratory experiments. Most examples have been described in digeneans [6-14], but there is also evidence from cestodes [15], monogeneans [16,17] and nematodes [18,19]. Testing whether or not hybridization may increase fitness by extending the range of suitable (intermediate) host species requires experimental studies to determine transmission success in the different stages of a parasite life cycle. Particularly in schistosomes, several studies have shown that hybrids between two species or strains inherited the ability to develop in both specific host snails of the respective parental lines and retain this increased host range over several generations [14,20,21]. Also for behavioral traits related to transmission, like diurnal cercarial shedding patterns [22] and specificity in host-finding behavior [23], hybrids of different *Schistosoma mansoni* strains have been shown to have trait values intermediate between the parental strains,.

*Schistocephalus solidus*, a cestode with a complex life cycle, is extremely specific for its second intermediate host, infecting only the three-spined stickleback *Gasterosteus aculeatus*. This system has become a model system for experimental studies on the evolutionary ecology of host-parasite interactions (reviewed by e.g. [24,25]). *Schistocephalus pungitii* is closely related to *S. solidus*, but uses the nine-spined stickleback *Pungitius pungitius* as second intermediate host, and shows the same host specificity at this level [26]. Both parasites potentially share the same final hosts [26] and often occur in sympatry [27,28]. Hence, natural encounters between adults of the sister species are plausible, making hybridization a possibility. However, a recent study by Nishimura and colleagues [29] shows a deep lineage divergence in the *Schistocephalus* genus, suggesting that separation of both species occurred shortly after the speciation of their respective stickleback lineages circa 20–25 million years ago. Hybrids have not been observed in nature yet and earlier experiments have shown that both *Schistocephalus* species are not able to infect the reciprocal intermediate hosts. Additionally, plerocercoids transplanted between three- and nine-spined sticklebacks stopped developing and later on showed destruction of the tegument [30,31]. Thus, these two species exhibit a high immunological specificity for their second intermediate host.

Many parasites undergo extensive growth in their final host, relative to that in their intermediate hosts [32]. However, *Schistocephalus* undergoes enormous growth in its second intermediate host. The worm is extensively challenged by the host’s immune system [33,34], so it is possible that this rapid growth is facilitated by highly specific adaptations to the host’s immune system. At least *in vitro*, the size of the worm is proportional to egg output [35,36], suggesting that specificity, growth, and fitness may be tightly linked in this system.

This system offers a unique possibility to investigate host specificity in two closely related parasite species with complex life cycles. It is likely that both parasite species meet in a bird’s gut for reproduction, which could facilitate interspecies mating. Both parasites are simultaneous hermaphrodites and capable of self-fertilization (selfing). Since selfing is costly for the parasite in all stages of its life cycle [37-40], hybridization would seem to be a good way to avoid the negative effects of inbreeding when outcrossing is not possible.

The aim of this study was to investigate the possibility of hybridization between the two cestode species of sticklebacks and the consequences of hybridization for host specificity and fitness at all stages of the parasite’s life cycle.

## Methods

### Study system

*Schistocephalus solidus* reproduces sexually in the intestines of piscivorous birds – their final host. Eggs are then released into the water with the bird’s feces, where they hatch into free swimming coracidia [41]. Copepods ingest coracidia and the worm develops into a procercoid in the copepod body cavity. When a three-spined stickleback feeds on infected copepods, the tapeworm is transmitted to its second intermediate host where it develops into a plerocercoid and undergoes enormous growth. The life cycle is completed when a piscivorous bird feeds on an infected stickleback [41]. *S. pungitii* shares the main characteristics of this life cycle, but uses *P. pungitius* as a second intermediate host.

**Figure 1:**
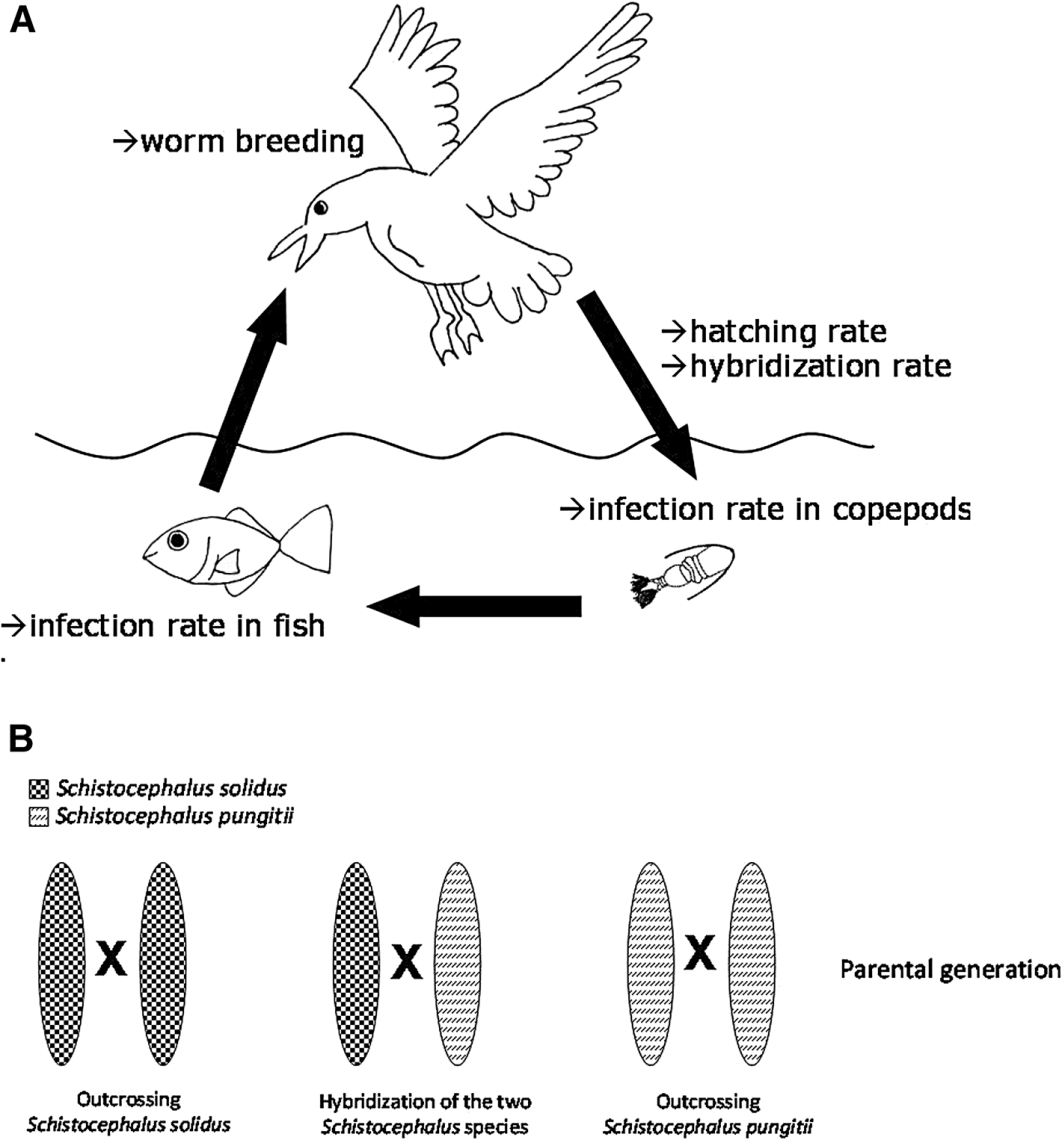
**Experimental design and measured parameters. A**: Life cycle of *S. solidus* and *S. pungitii* and parameters measured in this study. **B**: Experimental breeding design for hybrid worms.

The two species of parasite can be maintained in the lab for all stages of their life cycle. Plerocercoids are removed from the fish and can be bred in an *in vitro* system that mimics the bird’s gut [42,43]. Worms are usually size-matched for breeding, as this limits selfing [44]. After three weeks of incubation at 20°C in the dark, the coracidia start to hatch from eggs [26]. The coracidia can then be used to infect copepods (e.g. *Macrocyclops albidus*). After approximately two weeks of development in copepods, worms are infective to sticklebacks [45-47]. Figure 1 shows the life cycle of *Schistocephalus*, the most relevant traits measured in this experiment, as well as the breeding design for hybridizing the two parasite species.

### Breeding design and worm origin

Lab-infected sticklebacks originated from two allopatric populations: Skogseidvatnet, Norway (60°31^′^N, 05°13^′^E) for three-spined sticklebacks with *S. solidus* and Lebrader Teiche, Germany (54°22^′^N, 10°42^′^E) for nine-spined sticklebacks with *S. pungitii*. Fish were dissected and worms were paired for breeding. We used two different sibling families for each worm species (sibships, which refers to offspring from one pair of worms that were obtained from our lab cycle). For each sibship, at least one of the worms was paired with a conspecific from a different sibship, while at least one was paired with a worm from the other species. This was done so that the genetic composition of hybrid pairs and pure species pairs was similar, so that any observed differences between hybrid and pure groups are likely due to hybridization *per se* rather than random genetic differences between groups. Unfortunately, we were limited in the number of *S. solidus* plerocercoids, so we bred seven pairs in total: one outcrossed *S. solidus* pair, two outcrossed *S. pungitii*, and four potential hybrid pairs.

### Breeding conditions

The standardized laboratory breeding system [42,43] was slightly modified in that we diluted the medium with sterile filtered water, which we found was more suitable for *S. pungitii*. *In vitro* cultured, adult worms were transferred into netbags with their respective partner and these netbags put into a bottle containing pre-warmed medium (60% Eagle’s Minimal Essential Medium [Sigma] and 40% sterile filtered tap water). The bottles were incubated in a 40°C shaking water bath in the dark for two days. After two days we assumed that reciprocal fertilization had happened (see e.g. 36) and we isolated single worms in 50 ml tubes containing fresh pre-warmed medium. Eggs were collected from each worm for another three days in the breeding system. All collected eggs were washed with cold tap water (4°C) to prevent any larval development.

### Estimation of hatching rate & hybridization rate

The eggs were incubated at 20°C for 21 days in the dark. On day 21, the eggs were exposed to 4 h of light, followed by an 8 h period of darkness and another light period afterwards to stimulate hatching of coracidia. From each worm we aimed to collect 96 coracidia for determining hybridization rates via microsatellite analysis, while the remaining larvae were used to infect copepods. Low hatching rates limited the number of coracidia available in some groups (see Results). From the collected coracidia, DNA was extracted with chelex (after 44) and each individual was typed with microsatellites using six different loci (primers and PCR conditions in [48,49]) to estimate outcrossing/hybridization rates (see below).

The remaining eggs were left in a 16 hours light/8 hour dark room for another three weeks to ensure that every viable larva hatched. Afterwards, 100 eggs per worm were inspected visually to estimate the number of hatched coracidia.

### Exposure of copepods

Cultured copepods (*Marcrocyclops albidus*) (see [50] for details on cultures) were each exposed to a single coracidium. We aimed to expose 96 copepods per single worm, which could not be achieved in every case because of the low number of hatched coracidia in some worm sibships. The copepods were starved 1 day before exposure and afterwards fed every second day alternatingly with two *Artemia salina* nauplii or ~100 *Paramecium caudatum*. Copepods were checked visually for the presence of procercoids on day 8 and 9 post exposure. Infected copepods were then fed to fish on day 16. By this time, at least in *S. solidus*, worms are essentially fully developed and infective to fish [51], so infection success in fish is unlikely to be attributable to developmental variation [47].

### Infection of sticklebacks

Two German populations of naive lab bred sticklebacks (*G. aculeatus* from Großer Plöner See (54°07^′^N, 10°24^′^E) and *P. pungitius* from Lebrader Teiche (54°22^′^N, 10°42^′^E)) were used to test the infection success of hybrids and pure parental parasite lines in the second intermediate host. We used an allopatric combination for *S. solidus*/*G. aculeatus*, since we did not have access to enough fish from the sympatric population. Fish were put singly in plastic tanks containing approx. 1 L of water and starved for one day before exposure. Each fish was exposed to a single infected copepod. One day after the exposure the fish were moved in groups to 16 L tanks. The remaining water in the single tanks was filtered to ensure that all copepods were eaten by the fish. Fish were kept at 18°C and 16/8 h light/dark period and were fed three times per week *ad libitum* with frozen daphnids and chironomid larvae.

Nine weeks after exposure the fish were killed with an overdose of MS222, measured, weighed, and the body cavity was opened to remove and weigh worms if present. A tissue sample was collected from each worm for microsatellite typing to check whether it was an outcrossed, selfed or hybrid individual.

### Data analyses

We analysed the fitness relevant traits of the parasite separately. Two of the measured traits, hatching rates and outcrossing/hybridization rates, are characteristics of sibships and we analyzed them at this level. A generalized linear model (GLM) with quasi-binomial errors and a logit link function was used to compare hatching rates in the three groups (pure *S. solidus*, pure *S. pungitii* and hybrids). A similar GLM was used to compare the hybridization rate of hybrid pairs to the outcrossing rate of *S. solidus*. Only *S. solidus* and the hybrids could be compared, because, unfortunately, the microsatellite markers developed for *S. solidus* were not suitable to estimate outcrossing rate in *S. pungitii*, since all our individuals were homozygous across all loci.

Infection rates in copepods and fish were analyzed at the level of individual hosts. To evaluate whether infection rates differ between hybrids and the pure species groups, we fitted GLMs with binomial errors and a logit link function [52]. For infection rates in fish, in addition to the parasite group, we also included fish species (*G. aculeatus* and *P. pungitius*) as a factor. In some combinations of parasite group and fish species, no fish became infected (see Results). This kind of data structure (i.e. complete separation) causes inflated standard error and confidence interval estimates. Thus, we used the logistf R function (R package “logistf” [53]) to fit the GLM with penalized likelihood [54].

Finally, an analysis of covariance (ANCOVA) was used to test whether worm weight differs between fish species-worm species combinations while controlling for fish weight at dissection.

All statistical analyses were carried out using R 2.12.2 (R Development Core Team, Vienna). P-values lower than 0.05 were considered significant.

### Ethical statement

All animal experiments described were approved by the ‘Ministry of Energy, Agriculture, the Environment and Rural Areas’ of the state of Schleswig-Holstein, Germany (reference number: V 313–72241.123-34).

## Results & discussion

### Hybridization rate / outcrossing rate

Analysis of six different microsatellite loci revealed a hybridization rate of 24 to 49% in three hybrid pairs (a total of 141 typed coracidia). The remaining 76 to 51% were selfed individuals. The outcrossing rate in the *S. solidus* pair was 95% (91 coracidia typed). Both a Fisher’s exact test (P < 0.0001) and a GLM at the level of sibships (n=4, F_1, 2_= 24.04, P = 0.039) indicated that there were significantly more selfed individuals in hybrid pairs than in conspecific *S. solidus* pairs.

Our results show clearly that hybridization between *S. solidus* and *S. pungitii* from two allopatric populations is possible under laboratory conditions. The estimation of the selfing rate is biased by the fact that only hatched coracidia can be genotyped with microsatellites. The real rate of hybridization or outcrossing remains unclear since the genetic markers cannot be used on unhatched eggs [44].

### Hatching rate

A Fisher’s exact test indicated that overall hatching success differed significantly between groups (P = 0.0005). The hybrid pairs had the lowest hatching rate (mean 7.375%, n=4 worm pairs), followed by the *S. pungitii* (15%, n=2) and *S. solidus* (24.5%, n=1). However, the GLM with sibships as units did not indicate significant differences between the three groups (F-test comparing null model with model including treatment effect: F_2, 4_ = 1.72, P = 0.29). Thus, we conclude that hybrids tend to have lower hatching rates than pure species sibships, but a larger number of sibships must be observed to confirm this difference.

The lower hatching rate in hybrids may be a consequence of the strong inbreeding depression of selfed individuals, as it was also previously shown by Christen *et al*. [37] and Schjørring [40] that the hatching rate of selfed worms was about 4 to 8 times lower than in outcrossed individuals.

### Infection rate in copepods

**Figure 2:**
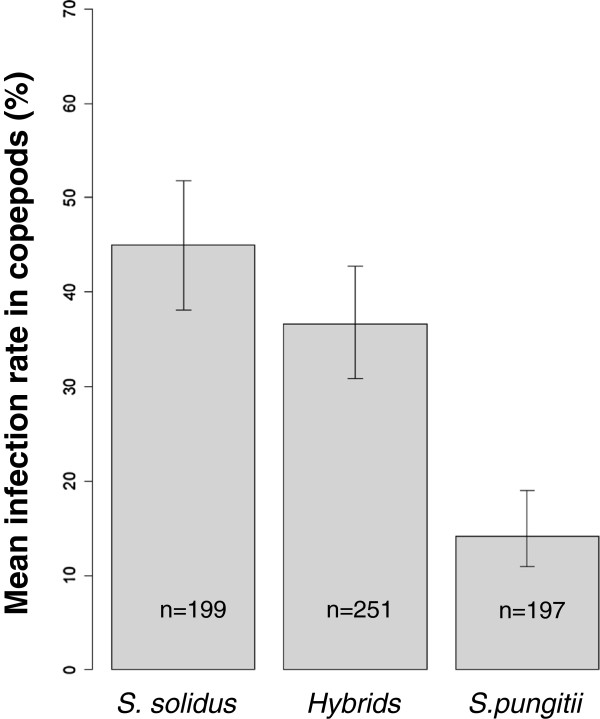
**Mean infection rate in copepods. **Error bars show 95% CI.

A GLM indicated significant differences between groups (likelihood ratio test with an intercept-only model, χ^2^_2_ =109.43, P < 0.001). Worms bred in hybrid pairings showed an infection rate that was between the infection rates of *S. solidus* and *S. pungitii* (Figure 2). Below we address the possibility that the relatively high proportion of selfed offspring in the hybrid pairs biases the infection rate estimate for hybrids.

### Infection rate in fish

**Figure 3:**
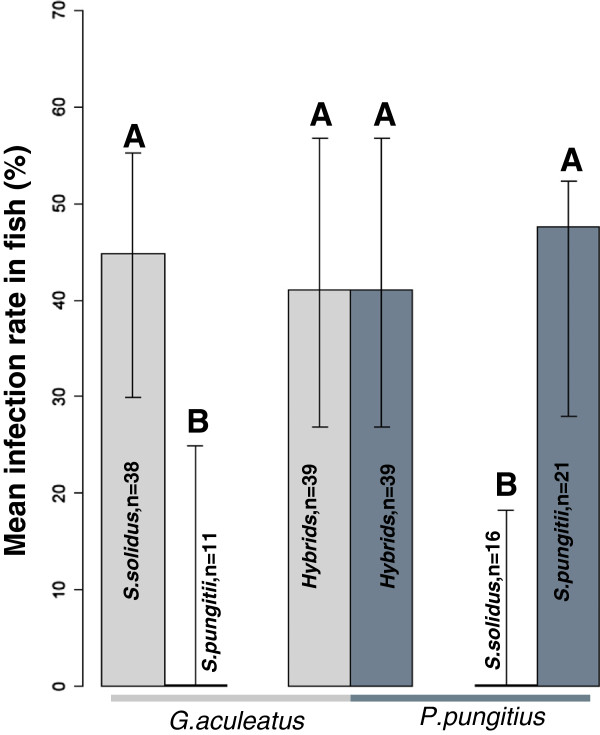
**Mean infection rate in fish. **Error bars show 95% CI. Groups with different letters (**A**, **B**) differ significantly from each other.

When we compare the infection rates in fish (Figure 3), we see that worm species or fish host alone doesn’t have an effect on the infection rate. This was supported by a likelihood ratio test that showed the GLM with an interaction term (fish species x worm group) was significantly better than the simpler model with just the two main effects (χ^2^_2_ = 25.41, P < 0.001). Microsatellite analysis after removal of the worms from fish showed that most worms from hybrid pairs were hybrids and not selfed individuals. In total, we found five selfed worms among 32 individuals. Again, the infection rate estimates for hybrids might be biased by the unknown proportion of copepods harboring selfed worms that were fed to the fish. We address this issue in the section below.

In our study, each species of worm was only able to infect its specific host while their non-host stickleback was never infected. Strikingly, the hybrids were able to infect both fish hosts with equal probability, while the pure lines only infected their specific fish host.

Since the hybrids are able to infect both fish hosts at similar rates, they have expanded their host range. If the genes responsible for this trait were purely additive, we would have seen ~20% infection rate of hybrids in fish (i.e. intermediate between the pure lines). Instead, we see a kind of co-dominance where hybrids can infect both fish hosts just as well as the parental lines. This ability may be due to specific traits that facilitate invasion and infection of both host species.

### Are hybrid infection rate estimates biased by selfing?

Eggs collected from hybrid worm pairs represent a mix of self-fertilized and hybrid offspring. Up to 76% of the coracidia typed from hybrid sibships were selfed, yet, of the plerocercoids recovered from fish exposed to hybrids, 27 were hybrids and 5 were selfed. Even though more selfed coracidia were presumably taken for copepod infections, hybrid worms were more likely to be recovered from fish at the end of the experiment. This suggests the estimated infection rates of hybrids in copepods and fish may be downwardly-biased; but how much? The observed infection rate in copepods, *R*_*c*_, equals:

$$R_{c}=\left(R_{\mathrm{ch}}*P_{\mathrm{ch}}\right)+\left(R_{\mathrm{cs}}*\left(1-P_{\mathrm{ch}}\right)\right)$$

where *R*_*ch*_ is the infection rate of hybrids in copepods, *R*_*cs*_ is the infection rate of selfers in copepods, and *P*_*ch*_ is the proportion of coracidia that are hybrids. The hybrid infection rate, our primary interest, thus equals:

$$R_{\mathrm{ch}}=\frac{R_{c}-\left(R_{\mathrm{cs}}*\left(1-P_{\mathrm{ch}}\right)\right)}{P_{\mathrm{ch}}}$$

**Figure 4:**
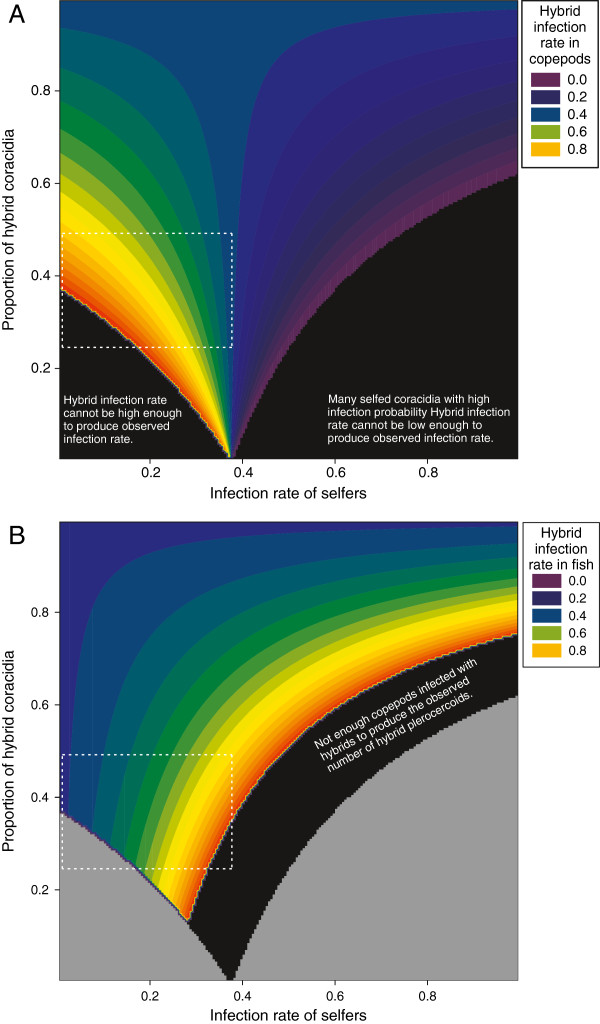
**Contour plots of the infection rate of hybrids in copepods (A) and sticklebacks (B) that reproduce the observed data. **Contours are plotted as a function of the infection rates of self-fertilized worms in copepods and the proportion of hybrid coracidia. See the main text for the equations used to calculate the hybrid infection rates. Black areas represent parameter space in which the observed results cannot be reproduced; intuitive explanations for this are given in each case. The gray areas in **(B)** are the black areas from **(A)**. Dashed white lines delineate the parameter space that we consider most plausible. The boundaries of this area on the y-axis were based on observed hybridization rates, which ranged from 0.24 to 0.49. The width on the x-axis was based on the assumption that, due to inbreeding depression, the infection rate of selfers is probably lower than the overall mean (0.375).

As *R*_*c*_ is known (=0.375), the infection rate for hybrids can be calculated for different combinations of *R*_*cs*_ and *P*_*ch*_. This is shown in Figure 4A. Inbreeding depression has been observed in *S. solidus*[37,40], so we may expect the infection rate of selfers to be lower than *R*_*c*_ (e.g. for the *S. solidus* population used here, other experiments determined the infection rate of selfed coracidia to be ~10%; D. Benesh, unpublished data). Moreover, the proportion of typed coracidia that were hybrids ranged from 24 to 49%. If we take these values to define a plausible range (*R*_*cs*_ < 0.375 and 0.24 <*P*_*ch*_ < 0.49), then Figure 4A indicates that the infection rate of hybrids in copepods may be substantially higher than estimated by the experiment.

This approach can be extended to calculate the infection rates in fish necessary to produce the observed number of hybrid plerocercoids. Assuming the proportion of selfed and hybrid worms infecting copepods are the same proportions used for the fish exposure (i.e. there is no differential mortality in copepods between the two groups), then the proportion of fish exposed to hybrid worms, *P*_*fh*_, equals:

$$P_{\mathrm{fh}}=\frac{R_{\mathrm{ch}}*P_{\mathrm{ch}}}{R_{c}}$$

The number of worms recovered from fish that are hybrids, *n*_*ih*_, is then, *n*_*ih*_ = *P*_*fh*_ * *n*_*e*_ * *R*_*fh*_,

where *n*_*e*_ is the number of fish exposed and *R*_*fh*_ is the infection rate of the hybrids in fish. Rearranging for *R*_*fh*_, our parameter of interest, gives Rfh=nihPfh*ne. *n*_*ih*_ and *n*_*e*_ are known (29 and 78, respectively) and *P*_*fh*_ is a function of the infection rate of selfers in copepods and the proportion of coracidia that are hybrids. Consequently, we can calculate the hybrid infection rate in fish necessary to produce the observed results, given different initial conditions (*R*_*cs*_ and *P*_*ch*_), and this is shown in Figure 4B. In the parameter space with the highest plausibility, hybrid infection rates were upwardly biased, but only slightly. Only with quite high selfer infection rates (>0.25) does this bias become large enough to suggest that hybrids have significantly higher infection rates in fish than the pure lines (>0.6).

In summary, our calculations indicate that the infection rate for hybrid worms in copepods may be much higher than estimated by the experiment, perhaps even higher than the pure *S. solidus* group (Figure 2). On the other hand, infection rate estimates in fish do not appear to be so biased that the rate for hybrids should be considered larger than that of the pure lines in their normal host (Figure 3). Thus, this analysis underscores our main conclusion; the hybrids do not experience any obvious fitness disadvantages compared to pure lines.

### Relationship between worm and fish body size

**Figure 5:**
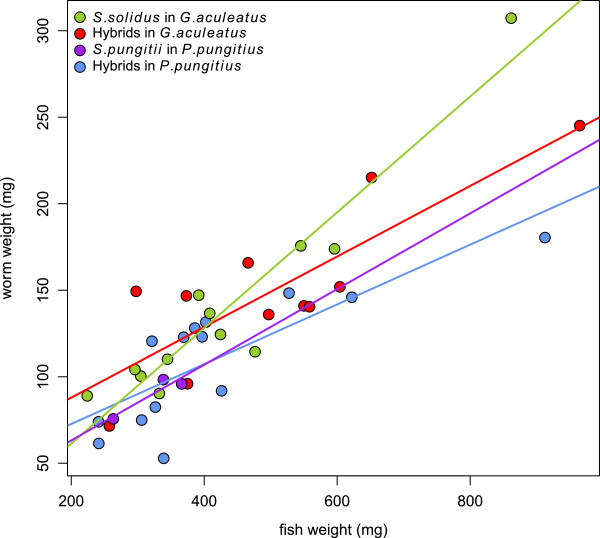
**Relationship between fish weight and worm weight. **The figure shows the relationship of fish and worm weight in mg for the different treatment groups. (*G. ac* with *S. solidus*: n=12, *G. ac* with hybrids: n=11, *P. pu* with *S. pungitii*: n=4, *P. pu* with hybrids: n=14).

There was a significant relationship between fish weight and worm weight (F_1, 39_ = 104.4, P < 0.001). Moreover, this relationship seemed to depend on the worm group (interaction between fish weight and worm group, F_3, 33_ = 8.68, P < 0.001) (Figure 5). Differences between groups were biggest in large fish, with pure *S. solidus* growing particularly large in *G. aculeatus* (Figure 5). Unfortunately, there were few data points in the largest fish, making these results tenuous. When we eliminated the data points from the largest fish (>0.7 g), there was no longer a significant interaction (F_3, 29_ = 0.09, P = 0.96) nor were there significant differences in the mean weight of hybrids and pure species worms (F_3, 29_ = 0.27, P = 0.84). Thus, these results suggest that hybrids and pure lines grow to quite comparable sizes in sticklebacks, although it remains possible that in larger fish worm sizes may diverge between groups.

Both the size of the host body cavity as well as its immune defenses are likely to limit worm growth. The host’s immune system is likely to interact with the parasite and interfere with its growth, as well as the space of the body cavity limits worm growth at a certain point.

## Conclusion

What is the evolutionary advantage of being highly host specific and why have no hybrid *Schistocephalus* been found in nature so far? There are several possibilities and none are mutually exclusive.

Ecological factors could cause prezygotic isolation between species. Both parasites species could have completely independent life cycles by inhabiting different microhabitats in the bird’s gut or even by infecting different bird species. Although *S. solidus* is known to be infective to a wide range of warm-blooded vertebrates [26,42], much less is known about *S. pungitii*. We also don’t know if the relatively high selfing rate observed in hybrid sibships is a consequence of the worms preferring to self instead of hybridizing or a consequence of a high proportion of unviable hybrids that did not hatch.

If the species are separated by postzygotic isolation, it may be that they hybridize frequently, but are either outcompeted by the pure lines or show a F2 hybrid breakdown [55-57]. We could show that at least the F1 generation of hybrids does not show obvious fitness disadvantages, which argues against the fact that they are rapidly outcompeted. Finally, barriers to hybridization may exist only in sympatric populations (reinforcement, for example see [58]). The *S. solidus* used in this study originate from a population in western Norway, where no nine-spined sticklebacks occur in the whole area (Per J. Jakobsen & Tom Klepaker, personal communication); therefore, in this specific situation there was no selection pressure to evolve a barrier to mating, which might be the case in populations where both stickleback species together with their specific parasites co-occur.

As other studies have shown, hybridization occurs in natural populations of different parasite taxa and has also been shown as a mechanism to broaden the host range by introgression of new genes [14,20,21].

It is worth noting that most studies on *S. solidus* and *S. pungitii* are based on morphological traits, which are not easily distinguishable between the species. To date, only a few studies have employed genetic markers [29,49] on a limited number of individuals, and therefore more extensive studies targeting the detection of hybrids are warranted.

Although we could observe hybridization in the laboratory, it still remains unclear if hybridization also occurs in nature. Further experiments are needed to test whether the worms are located in the same compartment of the bird’s gut, if hybridization can occur in natural hosts and if given the choice, worms choose mates of the same species over hybridization. Furthermore, it would be interesting to know how the possibility of hybridization and fitness parameters, such as infection rates in intermediate hosts, vary between sympatric and allopatric pairs of *S. solidus* and *S. pungitii* or if there even is a barrier to hybridization in nature. We are currently collecting more species pairs from different populations to test these ideas.

## Competing interests

The authors declare that they have no competing interests.

## Authors’ contributions

MK conceived the study. TH and MK performed the experiment, collected the data and wrote the manuscript. DPB performed the analysis for the evaluation of the infection bias and helped with the statistics and the manuscript. All authors read and approved the final manuscript.
